# Cue integration and the perception of action in intentional binding

**DOI:** 10.1007/s00221-013-3419-2

**Published:** 2013-02-01

**Authors:** Noham Wolpe, Patrick Haggard, Hartwig R. Siebner, James B. Rowe

**Affiliations:** 1 Department of Clinical Neurosciences, University of Cambridge, Herchel Smith Building, Cambridge, CB2 0SZ UK; 2 Medical Research Council Cognition and Brain Sciences Unit, Cambridge, CB2 7EF UK; 3 Institute of Cognitive Neuroscience, 17 Queen Square, London, WC1N 3AR UK; 4 Danish Research Center for Magnetic Resonance, Copenhagen University Hospital Hvidovre, 2650 Hvidovre, Denmark

**Keywords:** Volition, Intentional binding, Cue integration, Perception of action, Agency

## Abstract

‘Intentional binding’ describes the perceived temporal attraction between a voluntary action and its sensory consequence. Binding has been used in health and disease as an indirect measure of awareness of action or agency, that is, the sense that one controls one’s own actions. It has been proposed that binding results from cue integration, in which a voluntary action provides information about the timing of its consequences or vice versa. The perception of the timing of either event is then a weighted average, determined according to the reliability of each of these two cues. Here we tested the contribution of cue integration to the perception of action and its sensory effect in binding, that is, action and tone binding, by manipulating the sensory reliability of the outcome tone. As predicted, when tone reliability was reduced, action binding was diminished and tone binding was increased. However, further analyses showed that cue integration accounted for changes in action binding, but not tone binding. These findings establish a role for cue integration in action binding and support the growing evidence suggesting that action and tone binding are, at least in part, driven by distinct mechanisms.

## Introduction

The perception of the sensory consequences of one’s own actions is inherently different to the perception of other sensory events. For example, people tend to perceive the sensory consequences of their actions as attenuated (Blakemore et al. 1998; Shergill et al. 2003), which is proposed to facilitate the distinction between self- and externally generated actions (Blakemore et al. 2000). Another well-described perceptual distortion with voluntary actions is the temporal attraction between a self-generated action and its sensory outcome: A ‘willed’ action is perceived to occur later in time, whereas its sensory consequence (e.g., a tone) is perceived to occur earlier in time. This attraction is absent for involuntary actions, suggesting it is the intentionality that leads to the temporal binding of the action and its effect. The term ‘intentional binding’ is commonly used to describe this phenomenon (Haggard et al. 2002).

As intentional binding occurs only in the context of volitional actions (Haggard et al. 2002), it has been suggested to be a quantitative index of awareness of action or agency, that is, the sense that one controls one’s own actions. As an objective and replicable behavioral measure, it has considerable advantages over verbal self-reports in the study of volition. The intentional binding paradigm has therefore been applied to study agency in healthy individuals (e.g., Moore et al. 2011) and in clinical populations, such as individuals with Parkinson’s disease (Moore et al. 2010b) or schizophrenia (Haggard et al. 2003). In many of these studies, the magnitudes of ‘action binding’ (the temporal attraction of action toward its outcome tone) and ‘tone binding’ (the attraction of consequent tone toward action) are summed up to obtain an ‘overall binding’ measure. This measure is then interpreted as a positive correlate of agency. For example, the drug ketamine, which can induce a reversible psychosis in healthy individuals, enhances overall binding, similarly to that observed in schizophrenia, and has been suggested to increase agency (Moore et al. 2011).

Despite the growing use of binding as a measure of agency, the underlying mechanisms of action and tone binding remain largely unclear. Moore and Haggard (2008) have shown that action binding depends on both a predictive process (modulated by the probability of the tone following the action) and an inferential process (as action binding is apparent even in low effect probability as long as the tone occurs). Both of these processes are significantly supported by the contingency or causality relation between the action and tone (Moore et al. 2009), suggesting a critical role for learning an action–effect association. Tone binding on the other hand is related to a more general association process, as it does not depend on establishing a specific action–effect mapping (Desantis et al. 2012). A predictive process has also been suggested to account for tone binding, in which predicted sensory outcomes reach perceptual threshold more rapidly (Waszak et al. 2012).

Alongside these different accounts for action and tone binding, recent studies have shown specific modulations of either measure. For example, repetitive transcranial magnetic stimulation over the pre-supplementary motor area can specifically alter tone binding with no effect on action binding (Moore et al. 2010a). These studies suggest that action and tone binding may be driven by distinct mechanisms. Despite this body of evidence, there are few studies which examine the mechanisms of both action and tone binding. The present study aims to satisfy this experimental challenge by considering the role of cue integration in both action and tone binding.

In many sensorimotor tasks, perceptual phenomena have been successfully explained by cue integration models. These experimentally tractable models have also been suggested to contribute to the sense of agency, and the intentional binding in particular (Moore and Fletcher 2012; Moore and Haggard 2008). According to this framework, the sensorimotor system optimally combines information from different sources, such as multiple sensory modalities (Ernst and Banks 2002; Hillis et al. 2002) and prior expectations (Körding and Wolpert 2004), in order to reduce variability in performance (e.g., Ernst and Banks 2002). In binding, the action event and the sensory outcome event (tone) provide two separate cues for estimating their time. The time estimates are then a weighted average of the action and tone events, where the weight of each cue corresponds to its reliability (or in other words the precision of estimates, expressed as the inverse of the variance) relative to the reliability of the other cue. If both action and tone binding are supported by action–effect cue integration, this framework could explain the temporal attraction between action and tone events in binding.

In this study, we investigated the contribution of cue integration to action and tone binding. To this end, we manipulated the reliability of the tone event by modulating its intensity relative to a background white noise. Based on each subject’s individual auditory detection threshold, we generated three tones with increasing intensities, which in the presence of noise provided high, intermediate and low levels of uncertainty in the perception of tone onset.

We tested three main predictions of the cue integration hypothesis under different conditions of tone reliability. First, if cue integration underlies both action and tone binding measures, action binding will be weakest under high tone uncertainty, whereas tone binding will be strongest. These changes should be mainly driven by differential weighting of the action and tone cues according to uncertainty, in the conditions where both cues are provided. Second, if such cue integration mechanism is in fact common to both action and tone binding, the extent of changes in these measures as a result of modifying uncertainty will be related. Finally, in conditions where both action and tone cues are provided, the variability of time estimates should be lower (i.e., time estimates should be more precise) than in conditions where only one cue is provided, reflecting the key behavioral advantage of cue integration for perceptual precision.

## Methods

### Participants

Twenty right-handed volunteers (ten females) aged 18–36 (mean: 26, SD: 6) took part in the study and were compensated £14.5 for their participation. All subjects reported no history of neuropsychiatric disorders and had normal or corrected-to-normal vision. They all gave written informed consent before starting the experiment. The study was approved by the Cambridge Local Research Ethics Committee.

### Experimental procedure

Subjects were tested with a modified version of the intentional binding task (Haggard et al. 2002) using button press actions and auditory tone outcomes. Auditory stimuli were presented by Sennheiser HD250 Linear II headphones throughout the testing session. An auditory detection task was first performed to identify each subject’s detection threshold. White noise (1,000 Hz frequency) was played continuously, while pure tones (1,000 Hz; 100-ms duration) were played at intervals of 1–6 s. Tones were generated by multiplying the amplitude of a sinusoidal waveform by factors between 0.01 and 0.1 (fixed 0.01 interval between them). Overall level of noise was 80-dB SPL, and tones were between 63 and 83-dB SPL (intervals of 1–3-dB SPL between each tone). Subjects’ task was to press a key to indicate when they were able to hear a tone. For each tone intensity (10 in total), six trials were played pseudorandomly, making up a total of 60 trials. Stimuli were generated and presented using Matlab (Mathworks, CA) version 2009a.

**Fig. 1:**
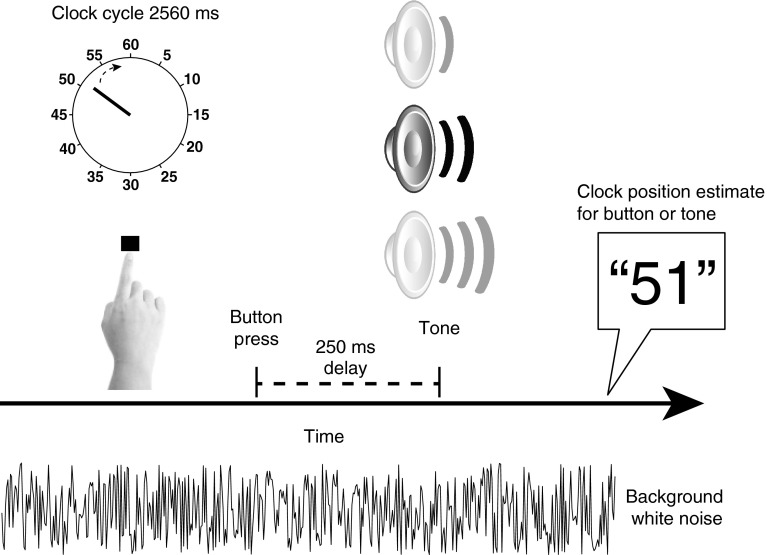
Illustration of the modified intentional binding task. Subjects attended to a ‘clock’ while hearing a background white noise. In the operant conditions, they were asked to press a button at their own pace, which triggered a tone (250-ms delay). The tone had low, intermediate or high intensity (interleaved in a pseudorandomized order). Subjects reported either the time of the button press or the time of the tone (conditions blocked) using the position of the rotating clock hand. Binding is measured as the difference between the means of estimation errors for ‘action’ or ‘tone’ events, and those in the corresponding baseline conditions, when the action and tone occur separately

On the main intentional binding task, subjects attended to a ‘clock’ on a computer screen marked with numbers from five to sixty in intervals of five (Fig. 1). A single hand rotated clockwise (period of 2,560 ms), providing a time stamp for reporting the perceived time of events. On each trial, the clock hand started at a random position. Subjects used a keyboard to report the time of self-paced button presses or tones (1,000 Hz; 100 ms). In the ‘baseline tone’ condition, a tone was played at random without a prior action between 2.5 and 6 s after trial onset. In the ‘baseline action’ condition, subjects made a button press, which was not followed by a tone. In the two operant conditions, a tone followed the button press by 250 ms, and subjects were asked to report *either* the time of their button press *or* the tone. These four conditions were blocked. On each trial, the clock stopped 1,500–2,500 ms after the event that was judged. Subjects were discouraged from pre-planning the time at which they press the button.

In contrast to previous intentional binding studies, a background white noise was played throughout the trials in order to increase the uncertainty about the time of tone onset. The tones had one of three amplitudes, generated as a function of each subject’s detection threshold (see “Analyses”). These three amplitudes were used to produce three levels of uncertainty with regard to estimating the time of tone onset. The three levels of uncertainty were pseudorandomly interleaved in the three task conditions in which tones were played. The task started with practice blocks. In the experimental blocks, each of the four block types consisted of 30 trials and was repeated four times. In total, 120 trials were performed for each condition, 40 trials per level of uncertainty. The four conditions’ blocks were presented in a counterbalanced order across subjects. The testing session was approximately 2 h long. All stimuli were displayed with Matlab Psychophysics toolbox (Brainard 1997).

### Analyses

The preliminary tone detection performance was fitted with a psychometric (Weibull) function, using a maximum-likelihood procedure (Wichmann and Hill 2001). Each subject’s amplitude of detection threshold was calculated at 50 % threshold in the psychometric function. In addition to the threshold amplitude, two more amplitudes were calculated, by multiplying that of the detection threshold by 2 and 5. This generated low (detection threshold), intermediate and high intensities for the tones used in the binding task. Across subjects, low tones had a mean intensity of 78-dB SPL; intermediate tones, 84-dB SPL; and high, 92-dB SPL; the noise was fixed at overall level of 80-dB SPL. Low, intermediate and high tone intensities were used to provide high, intermediate and low levels of uncertainty about the tone onset, respectively.

Mean estimation errors (i.e., the difference between actual and estimated time of event) were calculated separately for each level of uncertainty for action and tone in the baseline and operant conditions. Trials with outlier estimation errors (±2.5 SD from mean) were removed from each subject’s dataset (on average approximately three trials per subject). One subject was excluded from the study, as the standard deviation (SD) of his baseline action values was greater than two times the group mean SD. For each level of uncertainty, the mean estimation errors in baseline action and tone conditions were subtracted from their corresponding operant conditions to obtain action and tone binding measures, respectively.

To explore the effect of uncertainty on binding, we performed repeated-measures ANOVAs with uncertainty (high, intermediate and low) as a within-subject factor on the following datasets: (1) SDs of estimation errors in baseline tone condition; (2) action and tone binding values; and (3) mean estimation errors in baseline and operant tone conditions. Greenhouse–Geisser correction for non-sphericity was applied where appropriate. ANOVAs were followed by two-tailed paired t tests, except for the comparisons of binding across uncertainties, in which the direction was hypothesized according to the cue integration prediction. Two additional analyses were performed: (1) correlating the ratios between action binding in low and high uncertainty and the corresponding ratios in tone binding, using Spearman’s ranked correlation, and (2) pairwise comparisons of SDs in baseline versus operant action and tone conditions for each level of uncertainty. All pairwise comparisons were corrected for multiple comparisons using Bonferroni correction.

## Results

**Fig. 2:**
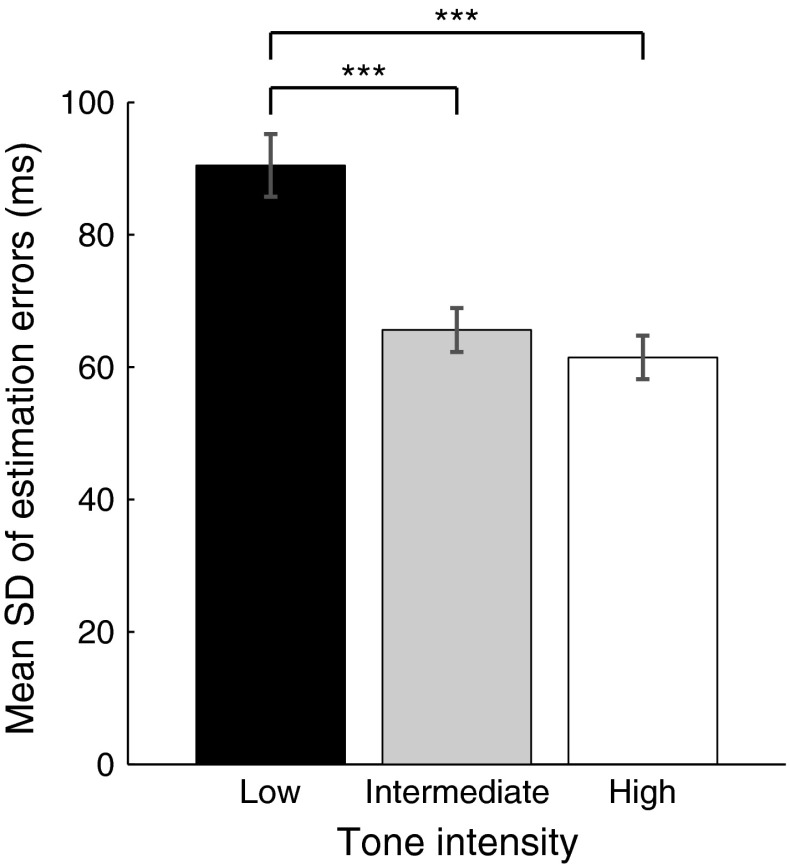
The standard deviation (SD) of estimation errors for the different tone intensities in baseline tone condition, wherein a tone was played at random without a prior action. SDs were increased in trials with low tone intensity relative to both intermediate and high intensities (***p < 0.001). High, intermediate and low tone intensities thus provided low, intermediate and high levels of sensory uncertainty in estimating the time of tone onset. *Error bars* indicate mean standard errors for the study group

Table 1 summarizes the estimation errors and their SDs for all experimental conditions across subjects. We first sought to verify the assumption that reducing tone intensities against the background noise would increase uncertainty with regard to estimates of tone event onset. We examined the SDs of estimation errors of tone onset in the ‘baseline tone’ condition, that is, where tones were played at random, and not associated with a button press or ‘action’ (Fig. 2). Repeated-measures ANOVA showed a main effect of intensity (*F*_(1.29, 24.43)_ = 23.8, *p* < 0.001) on estimation error SD. Post hoc two-tailed (Bonferroni corrected) comparisons confirmed an increase in the SD of estimation errors for low-intensity tones, relative to both intermediate (*t*_19_ = 4.38, *p* < 0.001) and high (*t*_19_ = 5.94, *p* < 0.001) intensities, and a weak trend toward increased SD in intermediate- compared to high-intensity tones (*t*_19_ = 1.69, *p* = 0.11). This confirms an increasing variability in time estimates with reducing tone intensity. High-, intermediate- and low-intensity tones were thus able to provide low, intermediate and high levels of temporal uncertainty, respectively.

**Table 1:** Mean estimation errors and mean standard deviation (SD) of estimation errors across subjects (mean standard error in parentheses). Values are shown for the estimated time of action and tone in the baseline and operant conditions for the three levels of uncertainty

Level of uncertainty	Condition	Event	Mean (SE) estimation error (ms)	Mean (SE) SD of estimation error (ms)
Low	Baseline	Action	−8 (11)	75 (6)
		Tone	35 (11)	61 (3)
	Operant	Action	31 (11)	79 (5)
		Tone	−16 (22)	76 (4)
Intermediate	Baseline	Action	−8 (11)	75 (6)
		Tone	46 (11)	66 (3)
	Operant	Action	23 (10)	70 (5)
		Tone	−19 (21)	80 (5)
High	Baseline	Action	−8 (11)	75 (6)
		Tone	95 (11)	90 (5)
	Operant	Action	24 (10)	71 (6)
		Tone	−10 (23)	77 (4)

**Fig. 3:**
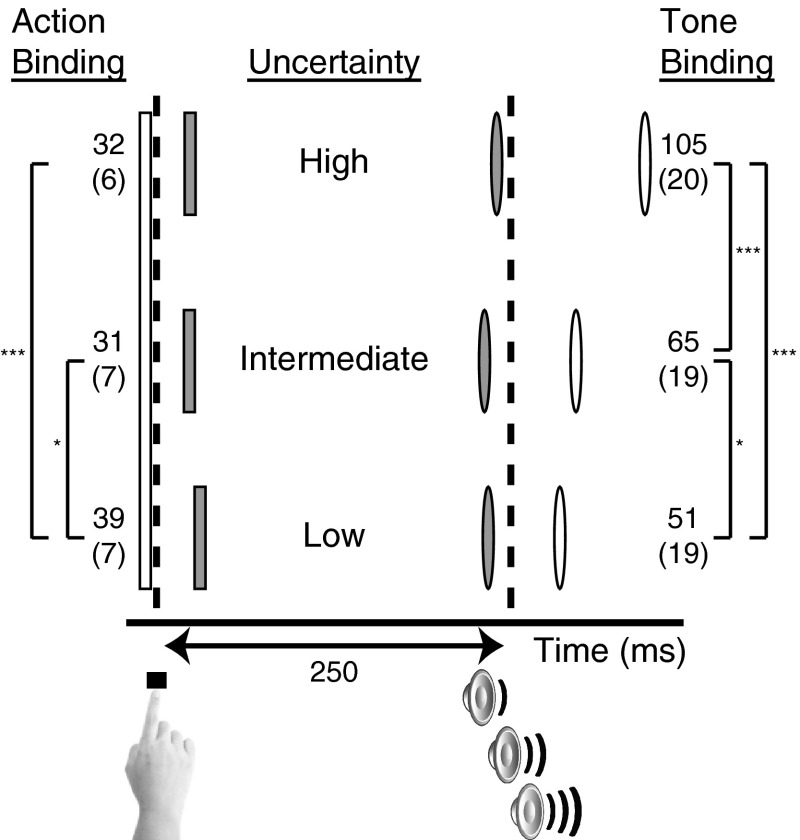
Action and tone binding for the three levels of tone uncertainty. *Dashed lines* indicate the veridical time of action and tone events. *Shapes* represent the event (*rectangle* action, *oval* tone), and their *shade* denotes the condition (*white* baseline, *gray* operant). All events are displayed proportionally to their time of occurrence. *Numbers* indicate the mean action and tone binding across subjects (mean standard error in parentheses). Significance level in pairwise comparisons is indicated by ***p < 0.001 and *p ≤ 0.05

The cue integration hypothesis predicts that as uncertainty about the timing of the tone increases (i.e., reliability is reduced), action binding will be reduced, whereas tone binding will be enhanced. To test this, we first calculated action and tone binding separately for each level of uncertainty, by subtracting estimation errors in the baseline conditions from their corresponding ‘operant’ conditions, in which the action was associated with a tone (Fig. 3). To examine the effect of cue integration on action and tone binding independently, we entered these measures to two separate repeated-measures ANOVAs, with uncertainty (low, intermediate and high) as a within-subject factor.

A main effect of uncertainty on action binding was found (*F*_(1.34, 25.37)_ = 4.22, *p* = 0.04). One-tailed (Bonferroni corrected) post hoc *t* tests indicated a reduction in action binding in the high relative to low uncertainty (*t*_19_ = −4.465, *p* < 0.001), and a just significantly reduced action binding in the intermediate relative to low uncertainty (*t*_19_ = −2.261, *p* = 0.05). A main effect of uncertainty was also found for tone binding measures (*F*_(1.64, 31.2)_ = 28.69, *p* < 0.001). Post hoc pairwise comparisons confirmed an increase in tone binding (i.e., an earlier estimate of the tone event) in the high uncertainty condition, relative to both intermediate (*t*_19_ = −5.546, *p* < 0.001) and low (*t*_19_ = −6.109, *p* < 0.001) levels of uncertainty. In addition, higher tone binding was observed in the intermediate compared to low uncertainty (*t*_19_ = −2.4, *p* = 0.04).

Differences in action binding could only result from changes in estimation errors in the operant condition, as there were no different levels of uncertainty in baseline action condition. By contrast, the differences in tone binding could arise from changes in either baseline or operant conditions, as estimation errors were calculated separately for each level of tone uncertainty in both of these conditions. To explore where differences in tone binding originated from, we examined the estimation errors in the baseline and operant tone conditions in two separate ANOVAs. A main effect of uncertainty on baseline tone estimation errors was found (*F*_(1.56, 29.7)_ = 69.81, *p* < 0.001), with significant post hoc (Bonferroni corrected) pairwise comparisons between the three levels of uncertainty (high–intermediate: *t*_19_ = 8.97, *p* < 0.001; high–low: *t*_19_ = 9.23, *p* < 0.001; intermediate–low: *t*_19_ = 2.88, *p* = 0.03). By contrast, there was no effect of uncertainty on operant tone estimation errors (*F*_(1.7, 32.31)_ = 1.88, *p* = 0.174). Taken together, these results indicate that the differences observed in tone binding across the three levels of uncertainty were driven by changes in estimation errors in the baseline condition, in which only the tone cue was provided. On the other hand, in action binding these differences could only arise from changes in the operant condition, indicating a contribution of cue integration.

We next examined the relation between changes in tone binding and changes in action binding across uncertainties. If the extent of change in action binding, arising from cue integration, is correlated with the extent of change in tone binding, this would support a common effect of cue integration on tone binding as well. Specifically, we looked at action and tone binding measures in the low and high uncertainty conditions (the conditions which showed a highly significant difference, above). We correlated the ratio between action binding in the low and high uncertainty conditions and the corresponding ratio in tone binding. There was no significant correlation between the change in action and tone binding as a result of uncertainty across subjects (Spearman’s rho = 0.038, *p* = 0.876), indicating there was no consistent relation between these changes in action and tone binding.

A critical assumption of the cue integration account is that the integration of multiple cues reduces variability in performance. Therefore, if cue integration contributes to tone binding, SD in the operant condition should be lower than the SD in the corresponding baseline condition. However, two-tailed (Bonferroni corrected) comparisons revealed a significant *increase* in SD in operant relative to baseline tone conditions in both intermediate (*t*_19_ = 3.92, *p* = 0.003) and low (*t*_19_ = 3.77, *p* = 0.004) levels of uncertainty. For action binding, the mean SD in operant action condition was lower than SD in baseline action condition for low and intermediate uncertainties, although not statistically significant (*t*_19_ = 1.66, *p* = 0.3; and *t*_19_ = 1.67, *p* = 0.3). Taken together, the results suggest that in contrast to action binding, tone binding is unlikely to be driven by action–effect cue integration.

## Discussion

We studied the contribution of action–effect cue integration to the perception of action and its sensory outcome in intentional binding. According to the cue integration hypothesis, the compression in the perceived temporal interval between a voluntary action and its sensory consequence results from using both events as temporally informative cues. The time estimates are based on a weighted average of the two events, in which the weight of each cue is determined by its relative reliability. As predicted by cue integration, our data show that reducing the reliability of the sensory outcome results in a smaller shift in the perceived time of action toward its outcome (reduced action binding). The cue integration hypothesis also predicted an increase in the shift of the perception of the sensory outcome toward the action with increasing uncertainty (i.e., increased tone binding). However, the results of additional analyses point to a separate mechanism involved in tone binding.

Action binding has been described in terms of a postdictive or inferential process, as it occurs even when the action is not strongly predictive of a tone, as long as the tone event occurs (Moore and Haggard 2008). Our results suggest that this postdictive process could be mediated by a cue integration mechanism. On the other hand, a predictive process has also been proposed to support action binding, as when the action is highly predictive of a tone, action binding occurs even in trials in which tones are absent (Moore and Haggard 2008). Consistent with this notion, even in our high uncertainty condition, in which tones were at each individual subject’s perceptual threshold, action binding measures were significantly above zero (data not shown). The association between the action and its outcome has been suggested to explain the predictive component of action binding (Moore et al. 2009).

Our finding that action binding is supported by cue integration is consistent with a previous study, suggesting that the estimation of time of movement depends on cue integration (Lau et al. 2007). This integration combines information about the time of action with other sources, as in the sensory outcome of an action. Information about the time of one’s own voluntary action could draw upon proprioceptive as well as internal volitional signals, such as an ‘efference copy’ of motor commands (Von Holst 1954) or components of the readiness potential (Lau et al. 2007). When sensory uncertainty is high or in the absence of sensory feedback, the perception of action relies more on these internal representations, thereby reducing action binding.

The cue integration framework has been successfully used to explain many perceptual phenomena in the sensorimotor system (e.g., Ernst and Banks 2002) and has been suggested to support the sense of agency (Moore and Fletcher 2012). Particularly, the integration of internal, volitional signals with external sensory cues can help dealing with uncertainty in the attribution of agency. Therefore, alongside the well-described action–effect association mechanism (see above), this integration could be another mechanism that links agency and intentional binding and reflects the volitional components that are captured in binding. For example, abnormal agency in disease states or under experimental interventions could arise from impairments in the internal volitional signals that normally contribute to the experience of agency. In turn, these impaired signals can lead to distinct changes in intentional binding, resulting from abnormal weighting of the action and outcome events. Future studies can thus apply the cue integration approach to explain abnormalities in action binding in terms of volitional deficits.

If cue integration can account for action binding effects, can it also explain tone binding? Our data suggest not. Tone binding was enhanced with increasing tone uncertainty, which at first glance is consistent with cue integration. However, this effect is attributable to increasing perceptual shifts in the baseline condition (tone only) as a function of tone intensity, rather than changes in the operant conditions, in which both action and tone events occurred together. Moreover, the changes in action and tone binding that resulted from sensory uncertainty were not correlated, suggesting different underlying mechanisms. Crucially, the prediction that integrating cues reduces performance variability was not satisfied for tone binding: Variability in estimation errors was significantly increased when two cues were provided in the operant tone condition, compared to baseline tone condition. These results show that the perceptual changes we observed in tone binding are likely to be driven by an alternative mechanism.

What might the alternative mechanism for tone binding be? Tone binding has been recently suggested to be mediated by a ‘pre-activation’ mechanism (Waszak et al. 2012). According to this account, the neural representation of a predicted sensory event, such as a sensory outcome following a voluntary action, is activated prior to its occurrence. Because of this ramped predictive activity, when the predicted sensory outcome occurs, it reaches perceptual threshold faster than when it is not predicted. Consequently, estimation errors are smaller in the operant tone condition than they are in the baseline tone condition, leading to tone binding. Our results suggest that this pre-activation mechanism can better account for the changes in tone binding under different levels of uncertainty. We found increased tone binding under high uncertainty, resulting from increased estimation errors in baseline tone condition. As tone intensity was reduced against a background noise for increasing sensory uncertainty, more time would be required for the tones to reach the perceptual threshold for detection. This additional time would be reflected in the increased estimation errors in the baseline condition. By contrast, in the operant condition, the learned action–effect association could diminish these differences in perceptual latencies. In other words, for pre-activated tone representations, the differences in intensities could be negligible, resulting in the lack of differences in estimation errors that was observed in the operant condition across uncertainties. Our results thus support the hypothesis that tone binding results from changes in perceptual latencies, driven by a predictive pre-activation mechanism (Waszak et al. 2012).

These results add to the growing evidence that different mechanisms underlie action and tone binding (Waszak et al. 2012). For example, whereas establishing a specific action–effect association is required for action binding (e.g., Moore and Haggard 2008), a more general association is sufficient for tone binding to occur (Desantis et al. 2012). In addition, experimental interventions can specifically affect tone binding without changing action binding. Such interventions include transcranial magnetic stimulation of the pre-supplementary motor area (Moore et al. 2010a) and manipulation of subjects’ causal beliefs (Desantis et al. 2011). Our results further support this notion: While action–effect cue integration is the most plausible explanation for the effect of uncertainty on action binding, differences in tone binding could be better accounted for by changes in perceptual latencies. Nevertheless, some mechanisms underlying action and tone binding may be shared. For example, the learned action–effect association can contribute to action binding through a prediction mechanism (Moore and Haggard 2008). Similarly, a prediction mechanism could be implemented for the tone to reach the perceptual threshold more rapidly and thereby lead to tone binding (Waszak et al. 2012).

Preliminary studies of the functional anatomy of binding (Moore et al. 2010a) have motivated the study of volitional disorders in patients with neurological and psychiatric illnesses, as well as healthy adults (e.g., Moore et al. 2010b, 2011). Often, action and tone binding measures have been added together (i.e., action binding plus the negative of tone binding) to generate an ‘overall binding’ measure. This measure has been used as a single metric of agency for comparing groups or measuring the effects of experimental interventions. However, if, as our data suggest, action and tone binding have different underlying contributory mechanisms, then disease states or interventions may have differential effects on these two forms of binding. Not only that our data indicate these two measures can be partially independent, but we also show that under some circumstances action and tone binding can be inversely related: Recall that high sensory uncertainty led to a reduction in action binding, while tone binding was increased. We therefore suggest that future studies should consider action and tone binding separately, rather than summing up these measures for studying volition.

The current study also has several limitations. First, our study draws upon the principles of cue integration, but does not apply computational techniques to model the data. Formal modeling of individual subject data would require many more trials for each condition per subject, which were not obtained here. Moreover, statistically optimal cue integration has been classically described for integrating multiple sources of information about one sensory event or object. Although action and tone events are synthesized in binding, it is possible that some of the principles of cue integration may not apply for the binding task, such as the statistical optimality. Second, we did not use a continuous variation of uncertainty, which would allow us to examine the psychometric properties of sensory uncertainty as a continuous effect. Such continuous variation was opted instead for the greater power conferred by the ordinal uncertainties. Third, our study only varies uncertainty in perception of action outcome tone and does not alter uncertainty in perception of time of action for fully covering the contribution of action–effect cue integration to binding. Manipulating temporal uncertainty of action would be experimentally more challenging. One solution could be to study clinical populations, such as patients with movement disorders, in which there is uncertainty over actions.

In conclusion, our results suggest that cue integration between action and effect contributes to the intentional binding effect for actions. By contrast, cue integration did not account for the observed changes in tone binding. This supports the notion that action and tone binding are driven by distinct underlying mechanisms. Our data support the use of intentional binding in the investigation of the mechanisms of volition, but suggest that action and tone binding should be considered separately in future studies.
